# Evaluation of a photographic food atlas as a tool for quantifying food portion size in the United Arab Emirates

**DOI:** 10.1371/journal.pone.0196389

**Published:** 2018-04-26

**Authors:** Habiba I. Ali, Carine Platat, Najoua El Mesmoudi, Mohamed El Sadig, Ihab Tewfik

**Affiliations:** 1 Nutrition and Health Department, College of Food and Agriculture, United Arab Emirates University, Al Ain, United Arab Emirates; 2 Institute of Public Heath, College of Medicine and Health Sciences, United Arab Emirates University, Al Ain, United Arab Emirates; 3 Division of Food, Nutrition and Public Health, University of Westminster, London, United Kingdom; 4 Department of Global Health and Population, Harvard T.H. Chan School of Public Health, Harvard University, Boston, MA, United States of America; State University of Rio de janeiro, BRAZIL

## Abstract

Although, United Arab Emirates (UAE) has one of the highest prevalence of overweight, obesity and type 2 diabetes in the world, however, validated dietary assessment aids to estimate food intake of individuals and populations in the UAE are currently lacking. We conducted two observational studies to evaluate the accuracy of a photographic food atlas which was developed as a tool for food portion size estimation in the UAE. The UAE Food Atlas presents eight portion sizes for each food. Study 1 involved portion size estimations of 13 food items consumed during the previous day. Study 2 involved portion size estimations of nine food items immediately after consumption. Differences between the food portion sizes estimated from the photographs and the weighed food portions (estimation error), as well as the percentage differences relative to the weighed food portion for each tested food item were calculated. Four of the evaluated food items were underestimated (by -8.9% to -18.4%), while nine were overestimated (by 9.5% to 90.9%) in Study 1. Moreover, there were significant differences between estimated and eaten food portions for eight food items (P<0.05). In Study 2, one food item was underestimated (-8.1%) while eight were overestimated (range 2.52% to 82.1%). Furthermore, there were significant differences between estimated and eaten food portions (P<0.05) for six food items. The limits of agreement between the estimated and consumed food portion size were wide indicating a large variability in food portion estimation errors. These reported findings highlight the need for further developments of the UAE Food Atlas to improve the accuracy of food portion size intake estimations in dietary assessments. Additionally, recalling food portions from the previous day did not seem to increase food portion estimation errors in this study.

## Introduction

The Arabian Gulf States (Bahrain, Kuwait, Oman, Qatar, Saudi Arabia, and the United Arab Emirates) have some of the highest prevalence rates of overweight, obesity, and other diet-related non-communicable diseases globally [1]. In the United Arab Emirates (UAE), cardiovascular disease remains the leading cause of death, accounting for 34.9% of all deaths in 2015 [2]. This highlights the need for locally relevant appropriate dietary assessment tools to determine energy and nutrient intakes of the UAE population. Accurate food portion estimation remains an important challenge in dietary recall assessments. Both under- and over-reporting of food portions consumed are major sources of error in the assessment of energy and nutrient intakes. This issue is even more critical in dietary recalls based on self-reports collected in nutrition surveys.

The most accurate method of estimating food portions consumed is weighing foods before and after consumption [3]. However, the weighed food method is time-consuming and has a high respondent burden, making it unsuitable for use in dietary surveys involving large numbers of individuals. Visual aids such as plastic food replicas (food models) and food photographs may minimize errors related to food portion size estimations in dietary recalls [4]. Food photographs may help improve the accuracy of food portion quantification in dietary recalls [4–6]. Moreover, previous research has shown that two-dimensional food photographs can provide food portion size accuracy similar to the three-dimensional food models [7]. The validity of using food photographs in portion size estimation has been studied in different countries and populations [5, 6, 8, 9]. The results of these studies support the usefulness of food photographs in quantifying food consumption portion size. However, since food habits vary across countries, it is essential for dietary assessments to use accurate food portion size estimation tools that are appropriate to the local context.

In preparation for a nutrition survey in the Emirate of Abu Dhabi, the Research and Development Division of the Abu Dhabi Food Control Authority recently developed the UAE Photographic Atlas of Food Portions (hereafter the UAE Food Atlas) and a companion user guide [10, 11]. The food atlas was designed to quantify food portions to assist research participants to estimate food portion size. It contains 115 foods commonly consumed in the UAE, Arabian Gulf and the Middle East Region. Of the foods included in the UAE Food Atlas, 82 were represented as a series of eight photographs each of increasing portion size. The remaining 33 foods were presented in a range of sizes similar to those normally available in the market. Details of food portion size selections used in the UAE Food Atlas have been previously reported [10]. Briefly, the weights of the foods identified to be common both in the UK and UAE were directly taken from the UK Food Atlas which were based on data from the National Diet and Nutrition Survey of British Adults [12].

The selection of portion sizes of foods not available in UK Food Atlas was based on food portions from a household survey that involved 26 UAE nationals, data on food portion sizes from a manual developed by the Kuwait Institute of Scientific Research [13] as well as input from experienced chefs. These foods were presented in 8 photographs in the UAE Food Atlas with 1/7^th^ of the difference in weight between the 5^th^ and 95^th^ percentile [10].

However, the validity of this newly developed tool in food portion estimation remains to be investigated. Therefore, there is a need to compare consumed food portions of known weights and volumes with food portion sizes estimated from the UAE Food Atlas before it can be adopted for widespread use in nutrition research and clinical practice in the UAE and elsewhere.

In the present study, we evaluated the validity of the UAE Food Atlas in estimating food portions consumed on the previous day by comparing with weighed food portions. Previous studies have reported memory recall and conceptualization as important elements that can affect food estimation errors from dietary recalls [5, 14, 15]. Thus, to minimize the effect of memory loss on food portion estimation errors, we also tested the accuracy of the UAE Food Atlas by comparing it with weighed food portions consumed by the participants on the same day.

## Materials and methods

### Study design and participants

Two observational studies were conducted to test the validity of the UAE Food Atlas compared with weighed foods. Study 1 (n = 132) involved previous day food portion size estimation and Study 2 (n = 65) food portion size estimation on the same day the food was consumed. Participants were recruited from a large national university in the UAE using a variety of methods, including face-to-face interviews, emails and telephone calls. In addition, to include participants with diverse demographic backgrounds (age, educational level and occupation), research assistants invited their family members to participate in the study. The exclusion criteria were age younger than 18 years and refusing to give informed consent for participation. Information about age, occupation, educational level and self-reported weight and height of the participants were collected.

### Ethical considerations

Ethical approval for the project was obtained from the United Arab Emirates University Human Research Ethics Committee (Protocol # 33; 2014/2015). Verbal and written informed consent were obtained from each participant.

### Choice of foods

A research team, which consisted of three students majoring in nutrition and three faculty members, reviewed the food items listed in the UAE Food Atlas. The criterion for choosing the food was that it must be represented in the UAE Food Atlas. The target number of food items to be tested was set to be at least 50% of the food items in the food atlas. Efforts were made to include a wide range of foods (different food groups, main dishes and desserts). Both UAE traditional and non-traditional foods in the UAE Food Atlas were considered for inclusion. The team identified a total of 65 food items to be included in the list.

### Study 1: Validation of previous day’s food portion size using photographic food atlas

Study 1 was conducted over two days. On the first day, research assistants observed the food selections of their family members at home and university students living in residential halls for one meal. Participants were included if they chose any of 65 food items in the list and informed consent was obtained. The research assistants weighed the serving containers that the participants used before the participant selected each food item. A food scale (Salter, Model SKU# 1047 HBBKDR14, Germany) was used to determine the food portion size before consumption. The serving container and the remaining food were weighed using the same scale after consumption, to determine the amount consumed. On the second day, survey participants were shown a printed copy or an electronic version of the UAE Food Atlas based on their preference. Participants selected the photographs representing portion sizes they considered to be closest to what they had consumed on the previous day. The research assistants recorded the names of food items consumed, the related food code listed in the UAE Food Atlas and the estimated and weighed food portion sizes. All research assistants attended a training about project protocols related to participant recruitment and data collection procedures.

### Study 2: Validation of food portion size consumed on the same day using the UAE Food Atlas

Study 2 was conducted on the campus of a large national university in the UAE. Nine commonly consumed food items were selected from the UAE Food Atlas with the aim of including more traditional food in the investigation. Food selection was limited to nine items due to logistical reasons (transportation of the food from restaurants to the study site and limited space to accommodate food items and participants). The food items were purchased from two restaurants that normally serve UAE traditional foods.

**Fig 1:**
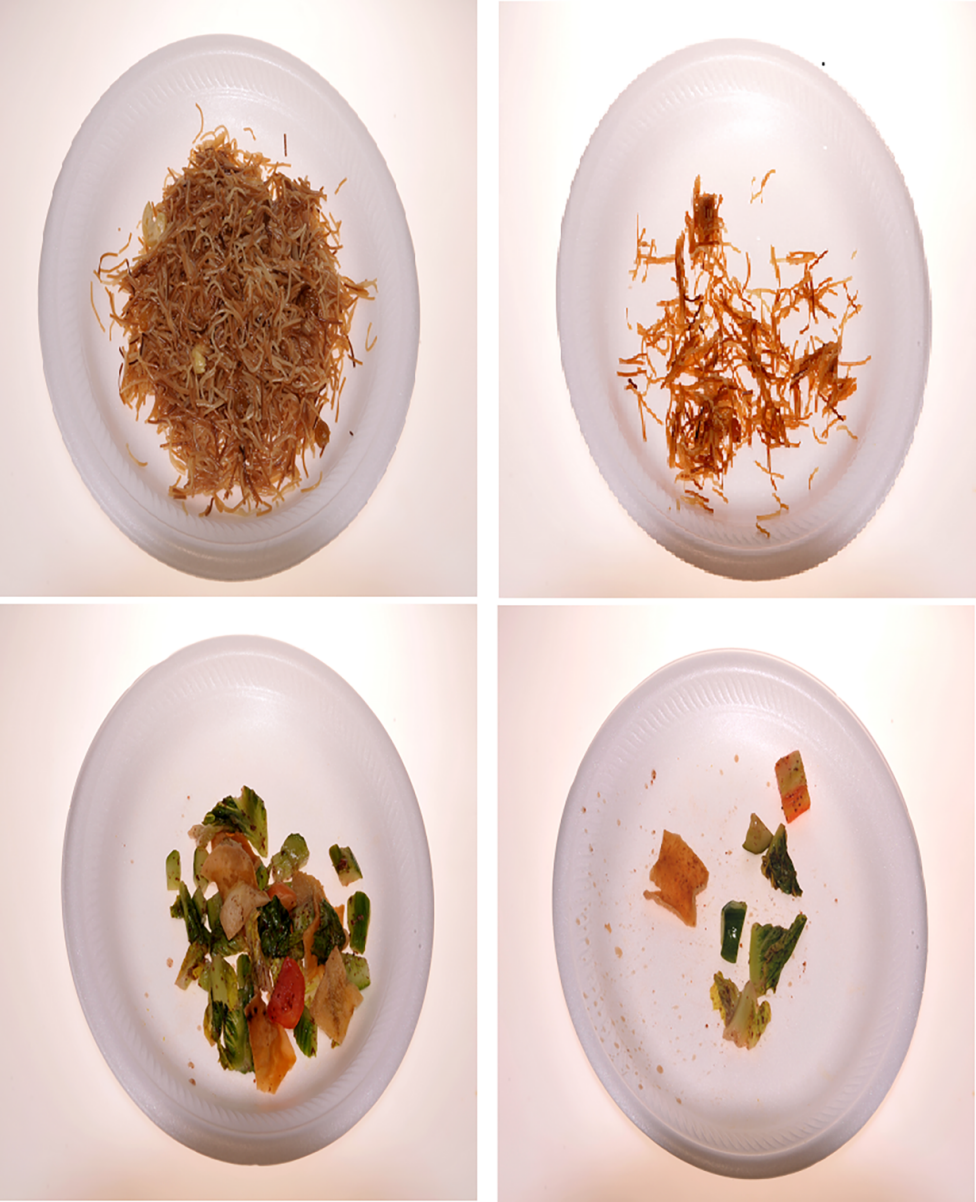
Examples of amounts of food before consumption and leftovers of the same meal. Amounts before consumption left, leftovers right.

After obtaining consent, participants were invited for a buffet-style meal. Participants served themselves in portions of their choices rather than being provided with standard portions. This approach was chosen to make food portion selections as close as possible to real-life eating situations. Participants were informed that they would be asked questions about the food, but not that they would be asked about food portions they consumed. Research assistants, using plastic plates with code numbers, weighed the serving containers before and after each participant selected their food. The food remaining in the serving container after consumption was also weighed to determine plate waste and thus the amount consumed. Monitors were present in the rooms to insure that different test foods were not mixed during eating and no food was discarded before re-weighing the plates. Pictures taken during Study 2 showing amount before consumption and leftover on the plate of two food items (green salad with bread and fried vermicelli) selected by one of the participants are presented in Fig 1.

Immediately after the meal, participants were shown photographs of portion sizes of the food items they had consumed, using a printed copy of the UAE Food Atlas. For each food item, participants selected the portion size they estimated to be closest to what they had consumed and the weight of the portion size, as listed in the food atlas, was recorded. The actual food portions eaten were rounded to the weight of the closest portion size represented in the UAE Food Atlas and also recorded. The rounded value was used in the subsequent analysis of the data.

Table 1 shows the main ingredients of the UAE and Middle Eastern dishes used in *Studies 1* and *2* with their corresponding UAE Food Atlas codes.

**Table 1:** UAE and Middle-Eastern dishes included in Studies 1 and 2.

Test Food	Test Food Local Name	UAE Food Atlas Code	Description
Spicy rice	Biryani rice	1	Spicy rice with vegetables
Rice with meat	Biryani meat	21	Spicy rice with meat and vegetables
Green salad with bread	Fattoush	51	Fresh vegetable salad with fried bread
Burghul parsley salad	Tabouleh	53	Burghul, parsley and tomato salad
Crushed wheat with meat	Harees	18	A thick porridge-type dish made with crushed wheat and meat and often served with ghee
Bread with meat stew	Threed	26	Stew-type dish made with meat or chicken, vegetables and traditional bread
Bread pudding	Ummali	74	Pudding made with puff pastries and nuts
Wheat pudding	Khabeesa	77	Traditional pudding prepared from Semolina, milk, nuts and spices
Fried vermicelli	Balaleet	79	Fried vermicelli

### Statistical analysis

Descriptive and comparative analyses of weighed and estimated food portions from the photographs were performed using IBM SPSS Statistics for Windows, version 23.0. Food portion estimation errors were determined by calculating mean differences between estimated and weighed food portions. A positive value indicated an ‘overestimation’ and a negative value an ‘underestimation’ of the food consumed. Food portion estimation errors were then expressed as percentage (%) of the food consumed. Histograms were used to check whether the differences between consumed and estimated portions were normally distributed which revealed that distributions were not normal.

Differences between the estimated and weighed food portion of each food item (estimation errors) were evaluated using Wilcoxon’s signed rank test to determine whether the estimation method significantly overestimated or underestimated the consumed portion sizes. Bland-Alman plots [16] were performed after grouping some of the food items to visually examine the agreements between the estimated and consumed food portions. The 95% limits of agreement and 95% confidence interval (CI) were calculated. For each variable, the mean of the differences (Estimated—Consumed) was plotted against the average of estimated and consumed portions means. Estimated and consumed food portions were converted into categorical variables to represent the image number in the food atlas corresponding to the estimated portion and the image number in the food atlas corresponding to the consumed portion. Weighted Kappa statistics [17] were used to determine the extent of agreement between these two variables.

A linear regression model was used to determine the associations between errors in food portion estimation and age, sex, body mass index (BMI–Kg/m^2^), and educational level. Since there was no hypothesis that a specific food item will have greater estimation errors than others, a subject’s estimation errors of the individual food items were pooled together by computing their root mean squared error (the square root of the mean of the squared estimation errors for each subject–the dependent variable). The dependent variable was regressed on gender, age, BMI and educational level (independent variables). P<0.05 was considered significant.

## Results

### Participant demographics

Table 2 shows the participant demographics. A total of 197 took part in the two studies. None of the participants took part in both studies. In both *Study 1* and *Study 2*, the majority of the participants were females (88.5% and 90.8%, respectively). Participants in *Study 1* were slightly older (mean age 27.4 ±11.18 years) than those in *Study 2* (mean age 25.1 ± 8.36 years). The educational level of the participants was relatively high. Only 6% of the participants in *Study 1* had less than a high school education and nearly 22% of participants in *Study2* had graduate level university education. The majority (68.7%) of the participants in *Study 1*were university students and the remaining were family members of the research assistants. Participants in *Study 2* were students and university faculty members (78.5% and 21.5%, respectively). According to self-reported weights and heights, nearly 38% of the participants in *Study 1* and 26% of those in *Study 2* were either overweight (BMI≥25.0 Kg/m^2^) or obese (BMI≥30.0 Kg/m^2^). The majority of the participants (77.3%) in *Study 1* used the printed copy of UAE Food Atlas to estimate their food intake portion size. Owing to participants’ time constraints, only the printed copy of the UAE Food Atlas was used in *Study 2* (i.e., 100% of participants).

**Table 2:** Participant demographics.

Characteristic	Study 1 (n = 132)	Study 2 (n = 65)
	n (%)	n (%)
Sex		
Male	15 (11.4)	6 (9.2)
Female	117 (88.6)	59 (90.8)
Age Group (yrs.)		
18–35	107 (81.1)	58 (89.2)
36–45	6 (4.5)	4 (6.2)
46–63	19 (14.4)	3 (4.6)
BMI Group (Kg/m^2^)		
Underweight	8 (6.0)	3 (4.6)
Normal (BMI:18.5–24.9)	74 (56.1)	45 (69.2)
Overweight/Obese (≥30.0)	50 (37.9)	17 (26.2)
Occupation		
Student	90 (68.7)	51(78.5)
Employed	29 (22.1)	14 (21.5)
Homemaker	7 (5.3)	0
Unemployed	5 (3.8)	0
Education		
≤ High School	8 (6.1)	0
Diploma	91 (68.9)	51(78.5)
Bachelor	27 (20.5)	0
Graduate studies	6 (4.5)	14 (21.5)

BMI–Body Mass Index (Kg/m^2^).

### Food portion size evaluations

Of the 65 food items in the original list, only 13 food items which at least 10 participants had consumed were included in the analysis of *Study 1*. The remainder were deemed insufficient in sample size to be included in the analysis. The mean estimated and weighed food portion sizes, as well as the mean differences and percent differences of the 13 foods tested in *Study 1* are presented in Table 3. Four of the food items were underestimated and nine were overestimated. Mean underestimates ranged from -8.9% for boiled rice to -18.4% for cucumber. Mean overestimates ranged from +9.5% for fries to +91.0% for spicy rice. Results from the Wilcoxon signed rank test showed significant differences (P<0.05) between estimated and weighed food portion sizes for eight of the food items tested in *Study 1* (Table 3).

**Table 3:** Estimated and weighed food portions consumed in the previous day (Study 1, n = 132).

Food item	Number of Estimations	Amount consumed (g)		Amount Estimated (g)		Difference (g)		% difference*	P-Value†
		Mean	SE	Mean	SE	Mean	SE		
Spicy rice	13	145.0	14.1	276.9	29.7	+ 131.9	20.4	91.0	0.002
Boiled white rice	34	167.8	15.0	152.9	11.4	-14.9	0.07	-8.9	0.084
Corn flakes	10	38.5	5.5	48.1	5.5	+9.6	7.20	24.9	0.202
French fries	36	95.9	9.0	104.9	7.0	+9.1	10.1	9.5	0.374
Meat curry	12	103.0	14.9	145.0	15.8	+42.0	16.35	40.8	0.039
Cucumber	21	43.9	3.4	35.8	3.0	-8.1	3.03	-18.4	0.023
Tomatoes	19	49.8	6.4	42.2	5.4	-7.6	2.72	-15.2	0.013
Coleslaw	10	88.5	18.3	124.5	16.5	+36.0	14.7	40.7	0.035
Green salad with bread	43	127.1	13.0	170.6	12.6	+43.4	10.5	34.2	0.001
Burghul parsley salad	10	94.1	16.3	81.5	13.8	+12.6	9.55	-13.4	0.144
Cream cheese	17	18.0	2.2	22.9	1.5	+4.9	2.0	27.4	0.022
Chocolate cake	14	88.4	10.2	114.9	13.5	+26.5	6.99	30.0	0.007
Soup	26	150.5	10.4	169.7	11.5	+19.2	13.52	12.7	0.128
Total number of estimation	265								

SE = standard error; (+) = overestimation; (-) = underestimation.

* % difference = [(mean amount estimated − mean amount eaten)/mean amount eaten] × 100.

†P<0.05 is considered significant (Wilcoxon’s Signed Rank).

In *Study 2*, of the nine food items tested, only bread with meat was underestimated (-8.1%). Overestimates were observed for the other eight food items (Table 4). Mean overestimates ranged from 2.5% for bread pudding to 82.1% for spicy rice. There were significant differences between estimated and weighed food portion sizes (P<0.05) for six of the food items. Fresh salad with bread was overestimated in *Studies 1* and *2* (43.4 g and 54.0 g, respectively). The largest mean percentage difference between the estimated and weighed food portion sizes was found for spicy rice in both *Studies 1* and *2* (+131.9 g and +87.7 g, respectively).

**Table 4:** Estimated and weighed food portions consumed on the same day (Study 2, n = 65).

Food item	Number of Estimations	Amount consumed (g)		Amount Estimated (g)		Difference (g)		% difference*	P-Value†
		Mean	SE	Mean	SE	Mean	SE		
Spicy Rice	32	106.8	4.6	194.4	20.1	+87.7	19.1	82.1	<0.001
Crushed wheat with meat	44	107.0	7.2	145.8	11.9	+38.8	9.6	36.2	<0.001
Rice with meat	23	125.1	11.8	199.2	25.4	+74.1	27.7	59.3	0.027
Bread with meat stew	27	178.8	59.9	164.4	17.4	-14.4	63.7	-8.1	.057
Green salad with bread	52	95.0	4.0	147.9	10.6	+54.0	97	55.7	<0.001
Fruit Salad	22	108.3	10.8	126.5	12.5	+18.2	8.9	16.8	0.058
Bread pudding	39	101.4	9.7	104.0	10.4	+2.6	117	2.5	0.976
Wheat pudding	18	45.1	2.3	65.3	10.5	+20.2	.9.6	44.7	0.031
Fried vermicelli	22	63.6	7.4	103.8	14.5	+40.2	12.3	63.1	0.005
Total number of estimations	279								

SE = standard error; (+) = overestimation; (-) = underestimation.

* % difference = [(mean amount estimated − mean amount eaten)/mean amount eaten] × 100.

†P<0.05 is considered significant (Wilcoxon’s Signed Rank).

**Fig 2:**
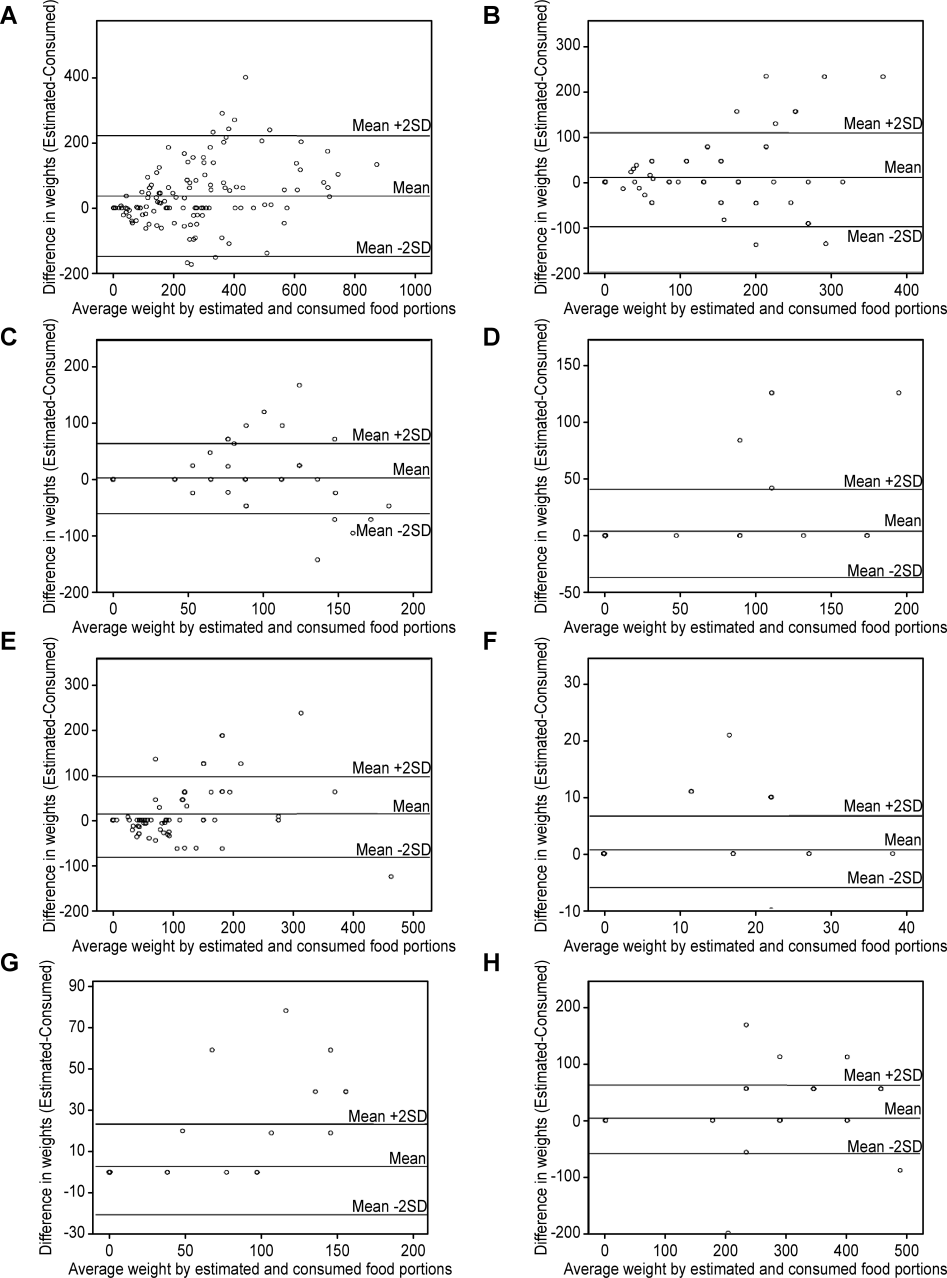
Comparison of estimated vs consumed weights (g) in Study 1. All foods (Panel A); Gains (Panel B); French fries (Panel C); Meat curry (Panel D); Vegetables (Panel E); Cream cheese (Panel F); Chocolate cake (Panel G); Soup (Panel H).

**Fig 3:**
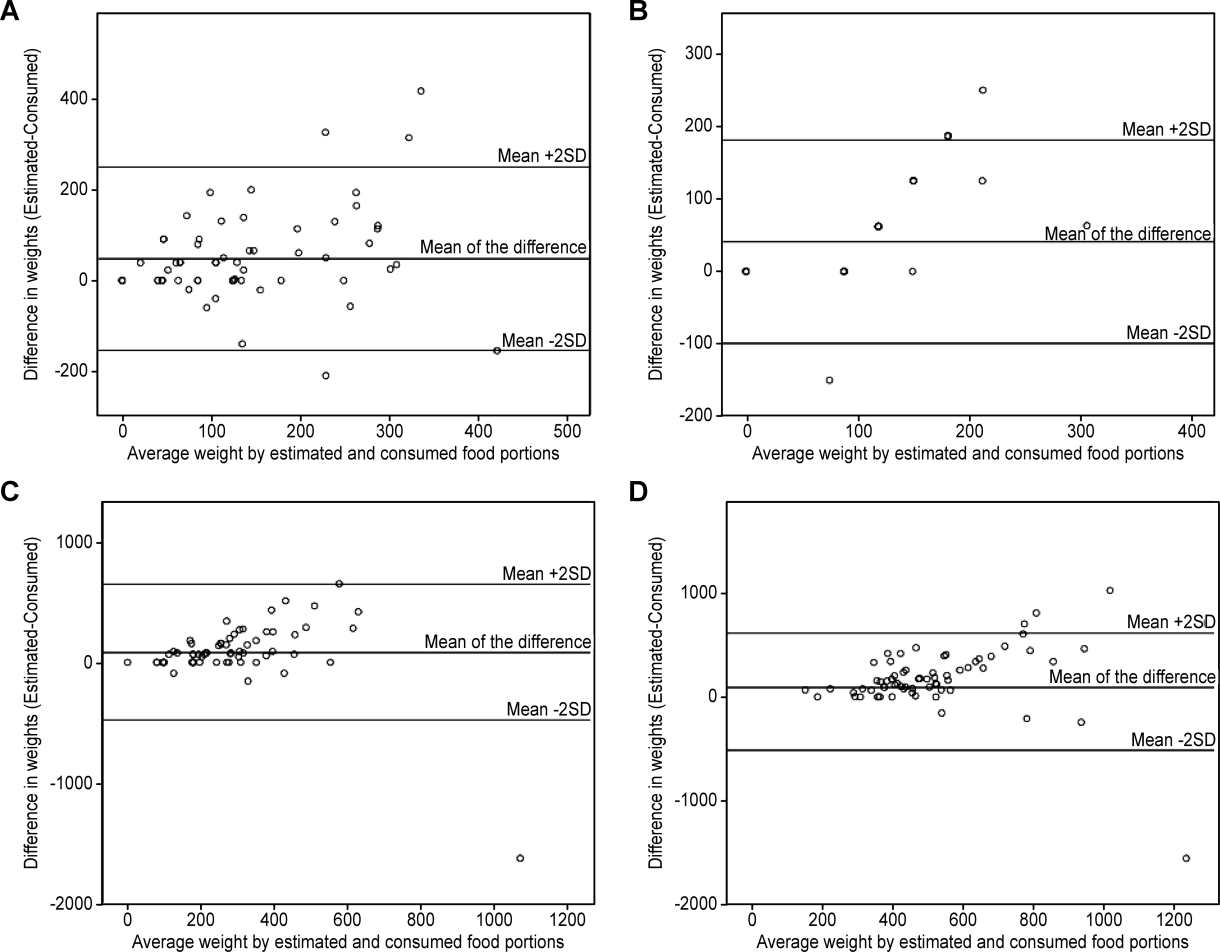
Comparison of estimated vs consumed weights (g) in Study 2. All foods (Panel A); Desert (Panel B); Green salad with bread (Panel C); Main course (Panel D).

Results from the Bland-Altman analysis indicated wide limits of agreement due to an increased dispersion with larger portions (Table 5). In both studies, the mean of the differences between estimated portion and consumed portion was slightly above zero (Figs 2 and 3). Based on the weighted Kappa (Table 6), a significant agreement is found for a great majority of food items in both studies, with a weighted kappa ranging from 0.243 for French Fries (p = 0.014) to 0.667 for tomatoes (p<10^−4^) in Study 1 and from 0.122 for green salad with bread (p = 0.016) to 0.462 for fruit salad (p<10^−4^) in Study 2.

**Table 5:** Bland-Altman limits of agreement and 95% confidence interval (CI) for the average difference between estimated and consumed food portions.

Food Item	Number of Estimations	Mean	S.D.	S.E.	95%CI	Limits of agreement	
						Mean-2SD	Mean+2SD
**STUDY 1**							
All food items	265	36.92	93.07	8.1	20.90–52.95	-149.22	223.06
Grain	57	9.87	54.48	4.74	0.49–19.25	-99.09	118.83
French Fries	36	2.47	31.58	2.75	-2.97–7.91	-60.69	65.63
Meat Curry	12	3.82	20.4	1.78	0.31–7.33	-36.98	44.62
Vegetables	103	13.55	48	4.18	5.28–21.81	-82.45	109.55
Cream Cheese	17	0.64	3.33	0.29	0.06–1.21	-6.02	7.3
Chocolate Cake	14	2.81	11.62	1.01	0.81–4.81	-20.43	26.05
Soup	26	3.77	31.09	2.71	-1.58–9.13	-58.41	65.95
**STUDY 2**							
All food items	279	60.62	304.83	37.81	-14.92–136.15	-549.04	670.28
Green salad with bread	52	40.86	70.14	8.7	23.48–58.24	-99.42	181.14
Main Course	126	89.63	263.03	32.63	24.45–154.81	-436.43	615.69
Dessert	101	47.92	101.15	12.55	22.86–72.99	-154.38	250.22

**Table 6:** Kappa agreement statistics for study 1 and study 2.

Study 1				Study 2			
Food Item	Weighted kappa	S.E.	p-value	Food Item	Weighted kappa	S.E.	p-value
Spicy Rice	0.071	0.051	0.220	Spicy rice	0.083	0.041	0.077
Boiled rice	0.592	0.081	<10^−4^	Crushed wheat with meat	0.394	0.103	<10^−4^
Corn flakes	-0007	0.149	0.969	Rice with meat	-0.045	0.093	0.707
French Fries	0.243	0.102	0.014	Bread with meat stew	0.245	0.123	0.027
Meat Curry	0.363	0.168	0.017	Green salad with bread	0.122	0.076	0.016
Cucumber	0.343	0.138	<10^−2^	Fruit Salad	0.462	0.095	<10^−4^
Tomatoes	0.667	0.094	<10^−4^	Bread Pudding	0.326	0.098	10^−3^
Coleslaw	0.824	0.167	0.093	Wheat Pudding	0.113	0.122	0.323
Green salad with bread	0.371	0.108	<10^−4^	Fried vermicelli	0.240	0.094	0.021
Burghul parsley salad	0.630	0.151	<10^−2^				
Cream Cheese	0.177	0.184	0.189				
Chocolate Cake	0.515	0.110	<10^−4^				
Soup	0.333	0.117	<10^−2^				

Results from linear regression analysis did not show significant associations between pooled error in food portion estimation and participant reported Body Mass Index (P = 0.958), educational level (p = 0.368), gender (p = 0.116) and age (p = 0.165).

## Discussion

Accurate recall of food portion size is critical in dietary assessment [18]. Food photographs have been suggested as a reliable tool for food portion size estimations in reports from the United States [19], France [20] and the United Kingdom [12]. Photographic food portion size tools can serve as an important tool in understanding population-based food consumption surveys in the UAE. We conducted two observational studies to evaluate the ability of the study participants to estimate food portions of commonly consumed foods using the UAE Food Atlas. Each food item in the UAE Food Atlas is composed of eight portions of increasing weight.

Our results showed that the majority of the foods tested in present two observational studies were either significantly underestimated or overestimated. The largest mean proportional percentage difference between the estimated and weighed food portions was found for spicy rice dish for both *Studies 1* and *2*. A consistent trend for underestimation or overestimation of specific food type, for example: fruits, vegetables, main dishes or sweets was not observed in this study, except for spicy rice. Results from Bland-Altman analysis showed that in most of the food items tested, large portions were associated with less agreement. Turconi and colleagues also reported a trend for decrease of agreement between intake and estimated food portion size as the intake portion size increases [8]

Previous research has shown that some foods are associated with greater estimation errors. A large variation in the accuracy of food portion size estimation was found in Mozambique dishes estimated by adolescents [21]. The largest mean percentage difference between the amount the estimated and the amount consumed was found for rice which was underestimated by 19%.

In Study 1, the Wilcoxon’s Signed Rank test for corn flakes was not significant (Table 3), however, this was not confirmed by the weighted Kappa. Although, the difference between the amount of corn flakes estimated and the amount consumed was low (9.6g) and was not significant, the standard error of the difference was particularly high (7.20). It is known that when the portion size is decreasing the impact of the error is increasing. For the other discrepancies, in terms of statistically significance, between the Wilcoxon’s Signed Rank Test and weighted kappa, both the agreement and the difference between the amount estimated and the amount consumed were significant. Again, the standard error of the difference was high which is indicating a low level of precision. Consequently, the probability to fall into the confidence interval of the difference is higher without an agreement.

Another study tested the validity of a food photograph album containing eight commonly consumed foods in Burkina Faso, presented in a series of four sizes of increasing weight for each food [9]. The reported correct estimation of the food portions was 55% of 1028 estimations. Significant estimation errors were found for three Lebanese dishes including burghul, parsley and salad [22]. In the present study, burghul parsley salad portion size was overestimated with estimation error of 13.4%, whereas in the Lebanese study, an underestimation error of 3.6% was reported. However, food photographs used in the Lebanese study were based on three food portion sizes compared with eight food portion sizes in the present study.

Several studies have found lower food portion size estimation errors from food photographs. The magnitude of errors in food portion size estimation using Lebanese food photographs ranged from -10.4 to +3.8 when the weighed food portions on the day before were compared with estimated food portions [22]. Among Italian dishes tested, reported errors in food estimation ranged from -2.7% for bread to +15.9% for vegetables [8]. Although food portion size estimation from food photographs can lead to both overestimations and underestimations [18], food photographs can still reduce errors in food portion estimations, compared with when estimation aids are not used [4].

Food portion estimation is a challenging task even when different food portion size estimation aids are used. Errors in food portion size of some individuals may reach 40% or more [15, 18, 23, 24]. The various elements that influence food portion size estimation, independent of the estimation aid used have been discussed [15]. Conceptualization of foods and memory while recalling amounts are important elements that can affect accurate recall of foods consumed [5, 15]. For example, participants must accurately perceive, conceptualize, remember and report the food portions consumed. Each of these elements can introduce errors in food portion size estimation. Hence, the purpose of Study 2 was to minimize the effect of memory as a contributing factor to variation in food portion estimation. However, recalling food portions consumed on the previous day is closer to the typical 24-hour dietary recalls used in individual dietary assessments.

In the present study, recalling food portion size immediately after consumption was not associated with lower food portion size estimation errors. This finding suggests that conceptualization is likely the main factor contributing to the food portion estimation errors in *Study 2*. The evaluation was conducted immediately upon food consumption, thus the effect of memory on food portion recall was minimized. In contrast, both conceptualization and memory could have contributed to estimation errors observed in *Study 1*, since the evaluation was based on recalling food portions consumed on the day prior to the interview. To the best of our knowledge, this study is the first to explore consumed food portion estimation based on previous day and immediately after consumption. We consider evaluation of food portion errors based on previous day food recall and immediate recall as a novel design and an important aspect of evaluating the UAE Food Atlas as well a valuable addition to the literature related food atlas validations.

Another strength of our study, to imitate real-life conditions of food selection. In *Study 1*, neither the food to be evaluated nor the portion size served were selected for the participants. In *Study 2*, participants served themselves in portions of their choices rather than being provided with standard portions.

It is common practice in the UAE for people to eat from a shared plate. This may create additional difficulties in the estimation of the individual food consumption from food photographs [25]. Also, measuring food ingredients during cooking is uncommon. These traditional habits may lead to additional challenges for the participants in this study to estimate food portions. In populations where measuring foods is not commonly practiced, accurate conceptualization of food portions might be lower leading to higher portion size estimation errors [26].

In both Study 1 and Study 2, there was a tendency to underestimate the food portion as reported by the participant. This is the case for all food items and food groups which were considered in this analysis. These results confirmed the previous differences described in Tables 3 and 4.

It is unknown whether using of fewer food photographs for each food item in the UAE Food Atlas would have improved the portion size estimation accuracy of the tested foods. Results from studies that examined less or greater number of photographs are conflicting. A series of three portion-size photographs used for food portion estimation in Lebanon and Italy were associated with relatively small errors [8, 22] In contrast, a large variation in the accuracy of the food portion size estimations was found for five Mozambique dishes evaluated based on three portion-size photographs [21]. A previous study reported better precision in food portion estimations using a greater number of photographs compared with using fewer photographs [23]. Similarly, an observational study that evaluated how best to provide digital images as portion-size estimation aids in the collection of 24-hour dietary recalls online, showed computer-based food photographs presented in eight vs four serial images had greater accuracy in food portion size estimation [15]. This might be partly explained by the fact that the photograph introduces an intrinsic error since it converts continuous portion sizes into ordinal level by asking participants to choose the closest portion size image to their food intake.

Although, use of food atlas is associated with intrinsic errors due to rounding of food portion sizes to the closest food photo by weight in the food atlas, this closely mimics real-life conditions whereby individuals are asked to estimate their food intake by comparing with the food portion sizes represented in a food atlas.

Higher proportion of food portion size underestimations among overweight participants has been previously reported [27, 28]. Previous studies reported inconsistent results of the association between educational level and food portion estimation accuracy [9, 24]. In the present study, none of demographic characteristics examined was significantly associated with food size estimation errors. However, this might be due to insufficient statistical power. Participants were free to choose their own food, leading a wide variety of foods consumed.

Food items presented in the UAE Food Atlas are not labeled with portion sizes. Currently there are no existing studies that have evaluated whether presentation of food weights along with the food photographs affects food portion recall accuracy. Thus, it might be useful to investigate the effects of food weight labeling on photographs in future investigations. One limitation of this study that affects the external validity of the results is that the majority of participants in this study were university students (68.7% in *Study 1* and 78.5% in *Study 2*). Therefore, further work is needed to evaluate the UAE Food Atlas with a larger sample size including participants from more diverse backgrounds.

The UAE Food Atlas is a convenient food portion size estimation aid and is appropriate in the local context. However, the results of this study report frequent over- and underestimation of food portion sizes whether food portion estimation was conducted based on foods eaten on previous day and immediately after consumption. It would be valuable to investigate whether minimizing the effect of the narrow range in weights of the eight portion size photographs that currently used in the UAE Food Atlas by using fewer number of portion sizes for each food item would improve the accuracy of the tool in food portion size estimation. Finally, previous studies have reported better accuracy at the group level [6, 8, 20].

Although the original list for *Study 1* included 65 food items, there were large variations of the food items consumed by the participants which made difficult grouping of the consumed foods into categories. Our data were collected in a natural setting where people choose their own food which led to greater variability of the foods consumed. Although some efforts were made in grouping certain food items together for the analysis of agreement tests, it was challenging to group food items together using shared characteristics based on either nutrient profiles or shape/form of the food. Therefore, additional investigations that involve larger numbers of food items categorized in groups, for example; fruits, vegetables, grains, mixed dishes and desserts should be investigated to evaluate food portion size estimation errors based on food groups.

On the other hand, an important strength of our study is to imitate real-life situations of food selection. In *Study 1*, neither the food to be evaluated nor the portion size served were selected for the participants. In *Study 2*, participants served themselves in portions of their choices rather than being provided with standard portions.

### Implications

In this study, estimating food portions from previous day was not associated with higher estimation errors compared to estimations performed immediately after food consumption.

Further investigations are needed to clarify whether time lag has a minimal effect on food recall portions or the estimation errors are mainly due to poor conceptualization of the images in the food atlas regardless of the lag time.

### Conclusions

Evaluation of previous day and immediate food consumption based on UAE Food Atlas appears not to affect the accuracy of food portion size in this study. The results of our study highlight the need for further improvements of the UAE photographic food atlas before adopting it for widespread use in nutrition research and clinical practice in the United Arab Emirates and elsewhere in the region.

## Supporting information

S1 File Evaluation of UAE Food Atlas Study 1 dataset. (XLSX)

S2 File Evaluation of UAE Food Atlas Study 2 dataset. (XLSX)

S3 File Local food names used in Study 1 and Study 2 datasets. (XLSX)
